# Case Report: Large mediastino-abdominal hydatid cyst extending through the Larrey's hiatus: A rare case report

**DOI:** 10.12688/f1000research.159463.1

**Published:** 2024-12-05

**Authors:** Houssem Messaoudi, Habib Bessrour, Mokhles Lajmi, Wafa Raghmoun, Wael Ferjaoui, Mohamed Bechir Khalifa, Saber Hachicha

**Affiliations:** 1 cardio-thoracic department, Military Hospital of Instruction of Tunis, Tunis, Tunis, Tunisia; 2 General surgery department, Military Hospital of Instruction of Tunis, Tunis, Tunis, Tunisia

**Keywords:** hydatid cyst, liver, mediastinum, Larrey's hiatus.

## Abstract

**Background:**

The extension of hydatid liver cysts into the mediastinum through diaphragmatic hiatus is extremely rare. In this report, we describe a rare case of a hydatid liver cyst with mediastinal extension through Larrey’s hiatus, emphasizing the surgical strategy for successful treatment.

In this report, we present the first documented case of a hydatid liver cyst extending into the mediastinum through Larrey’s hiatus.

**Case presentation:**

We report the case of a 65-year-old male who presented with right-upper-quadrant and left-sided chest pain evolving for two months. Physical examination showed dullnes in the epigastric region. A thoraco-abdominal CT scan, revealed a 13 cm mediastino-abdominal hydatid cyst, centered on the left liver lobe with extension into the mediastinum through the Larray’s diaphragmatic hiatus. The patient underwent surgical management. Both thoracic and visceral surgeons were involved. The surgical management involved both thoracic and visceral surgeons. A Makuuchi incision allowed resection of the hydatid liver cyst. The mediastinal portion of the cyst, resting on the pericardium was aspirated through Larrey’s hiatus, followed by irrigation with a scolicidal solution. The postoperative course was uneventful.

**Conclusion:**

Transmediastinal hydatid liver cysts are rare and should be operated on in close collaboration between visceral and thoracic surgeons to prevent complications.

## Introduction

Hydatidosis is an anthropozoonosis due to the development of the larval form of *taenia echinococcus granulosis* in humans. ^1^ Echinococcal cysts involve predominately the liver (60–70%) and the lungs (15%). It arises in other abdominal organs in 10% of cases. ^2^ While the hepatic involvement is well-documented, cases of hydatid liver cysts extending into the mediastinum through a diaphragmatic hiatus are exceedingly rare. ^3^

This unusual presentation poses unique diagnostic and therapeutic challenges, requiring a multidisciplinary approach for optimal management.

We report herein a rare case of a 65-year-old man with a hydatid cyst involving the liver and the mediastinum with anatomical continuity through the Larry’s hiatus.

This case highlights the importance of a well-planned surgical approach and close multidisciplinary collaboration for successful intervention.

## Case presentation

**Figure 1.**
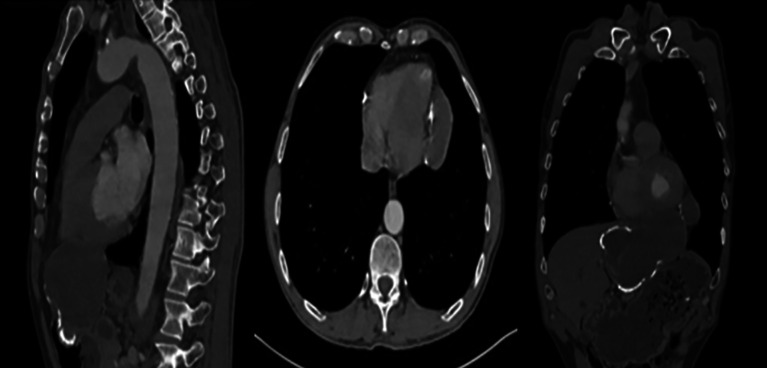
CT scan showing the hydatid liver cyst passing through Larrey’s hiatus.

A 65-year-old male patient, without past medical history, presented with right upper quadrant pain, associated with left-sided chest pain and epigastric discomfort evolving for two months. Physical examination showed dullnes in the epigastric region. The laboratory findings were normal. Chest radiography revealed a mediastinal widening. Abdominal sonography revealed a large type IV hydatid cystic, necessitating further characterization through a computed tomography (CT) scan. Serological tests detected the presence of anti-hydatid cyst antibodies,

Computed tomography showed a 13 cm cystic mass centered on the left lobe of the liver, extending into the mediastinum through the Larry’s hiatus, coming to rest intimately on the pericardium with broad contact ( Figure 1). Furthermore, a calcified cyst was noted in the spleen.

**Figure 2.**
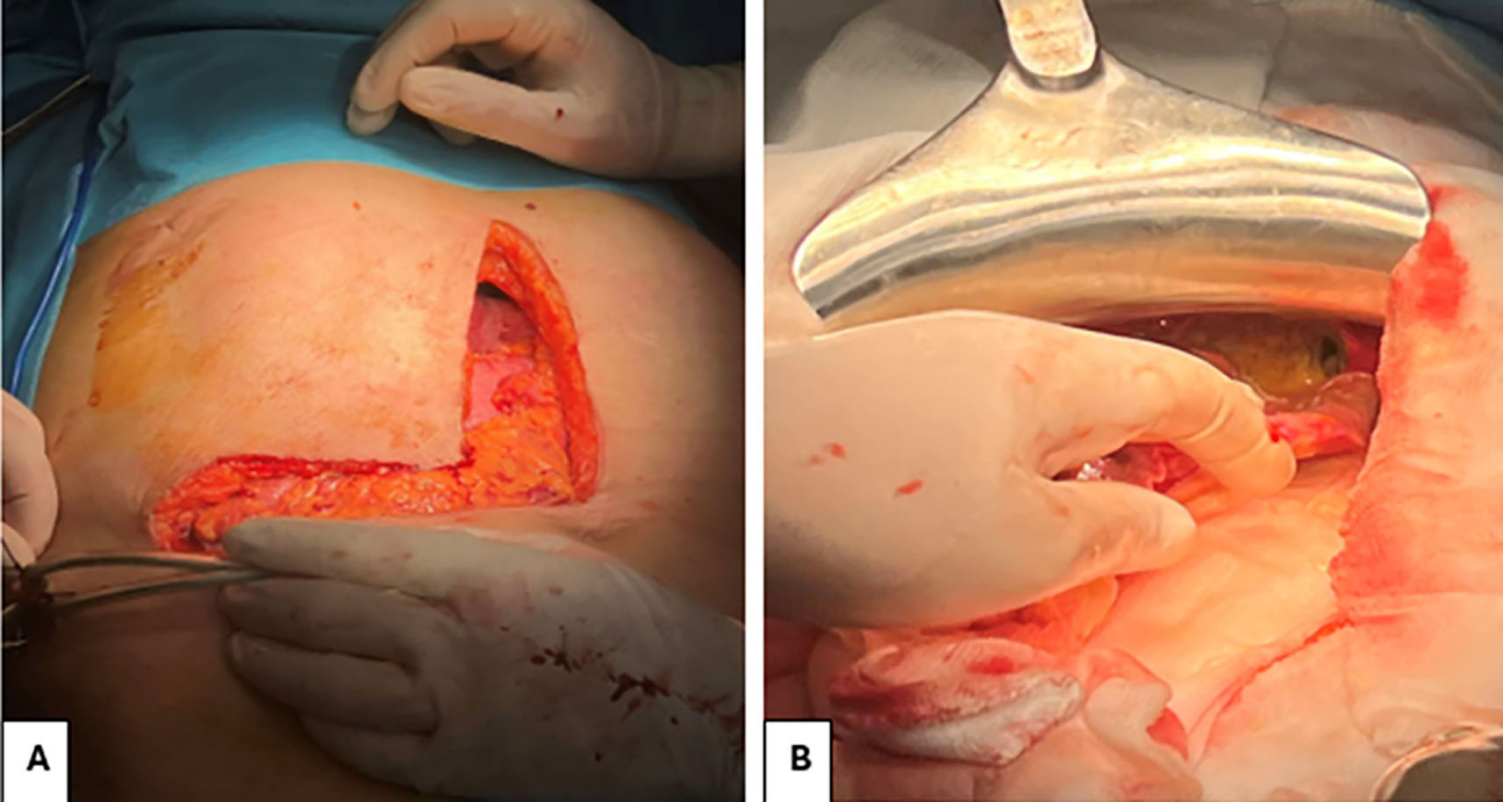
A: Surgical route: Makuuchi incision B: Intraoperative image showing the orifice of the mediastinal extension of the hydatid cyst.

The patient underwent surgery performed by a dual team of thoracic and visceral surgeons. The approach involved a Makuuchi incision ( Figure 2). During exploration, a large hydatid cyst was identified at the hepatic dome with mediastinal extension through Larrey’s hiatus. Resection of the protruding dome was performed, followed by aspiration of the hydatid fluid. An aspiration catheter was introduced through the orifice of the transmediastinal extension to evacuate the hydatid content. An irrigation with a scolicidal solution was performed. The procedure was concluded with an omentopexy into the remaining cavity. The calcified hydatid cyst of the spleen was preserved.

The postoperative course was uneventful. The patient was discharged well on oral albendazole with a posology of 400 mg per day for six months. No recurrence was observed after one year of follow-up.

## Discussion

Hydatid disease remains a significant public health concern in Mediterranean countries such as Tunisia. ^1^ The most frequent location are the liver and the lung. The mediastinal location is extremely rare, with anecdotal reports in the literature accounting for less than 1 % of cases. ^4^ In the majority of cases, the mediastinal hydatid cyst is unique. ^5^ The simultaneous involvement of the liver and mediastinum by a single hydatid cyst is an extremely rare condition. ^3^ A classification was established by Gomes et al. on the transdiaphragmatic extension of hydatid liver cysts, but it did not specify the extension through diaphragmatic hiatus. ^6^

To the best of our knowledge, this exceptional case of hydatid liver cyst extending to the mediastinum through Larry’s hiatus, represents the first ever documented in the literature, underscoring its extreme rarity. Lahdhili M reported a case of a gigantic mediastino-abdominal hydatic cyst extending through esophageal hiatus. ^3^

The physiological thoraco-abdominal pressure gradient suggests that the cyst likely originated primarily in the liver, with its anterior mediastinal extension occurring through a weakness in the anterior diaphragmatic muscle, which must be the Larrey’s hiatus. Most patients with mediastinal hydatid cyst are symptomatic. ^7^

The presenting symptoms depend on the cyst’s size, location, and the extent of compression or erosion of adjacent mediastinal structures. In our case the patient presented with a two month right upper quadrant pain, associated with left-sided chest pain and epigastric discomfort. Mediastinal hydatid cysts can lead to serious complications, such as rupture, fistula development, infection, and pressure on vital structures. ^7^

Imaging investigations play a major role in the detection of hydatid liver cysts. The diagnostic imaging evaluation is based on multiple modalities imaging, including ultrasonography, computed tomography (CT) and magnetic resonance imaging (MRI). In our case, the diagnosis was made based on the sonographic findings and scan data.

The differential diagnoses may include cystic lymphangioma, enteric cyst, bronchogenic cyst, and pleuro-pericardial cyst. ^8^

Surgery remains the optimal treatment modality of hydatid liver cyst, typically involving resection of the protruding dome. However, the mediastinal extension through Larry’s hiatus made the procedure more challenging, requiring close collaboration between thoracic and visceral surgeons. The standard treatment for mediastinal hydatid cysts typically involves pericystectomy, which includes the removal of the germinal membrane via a thoracic approach. In cases where the cyst’s localization and its invasion of vital structures complicate total excision, partial pericystectomy is recommended after the germinal membrane has been removed. ^5^ For liver hydatid cysts, an abdominal approach can be effectively employed, even when there is transdiaphragmatic extension into the thoracic cavity. ^6^

In our case, we opted for an abdominal approach, starting with the resection of the protruding dome of the cyst, followed by aspiration of the hydatid fluid through a Makuuchi incision.

The primary objective was to minimize surgical risks when dealing with cysts closely adhering to critical structures such as the esophagus or pericardium. Alternative strategies may involve solely performing a thoracotomy or combining both thoracotomy and laparotomy in a single surgical session. ^9^

Our decision to take this route was to address the hydatid cyst of the liver while also assessing for any potential biliary fistula. This approach allowed us to aspirate the cyst contents from the mediastinal side and inject a scolicidal solution, aiming to manage the infection effectively while minimizing the risk of complications.

## Conclusion

Surgical intervention remains the most effective treatment to prevent complications associated with hydatid cysts. The extension of hydatid liver cysts into the mediastinum through the diaphragmatic hiatus is indeed an atypical presentation that requires careful planning. This scenario often necessitates collaboration with visceral surgeons to determine the optimal surgical access route and technique.

In addition to surgical management, complementary adjuvant pharmacological therapy is essential to reduce the risk of recurrence. This combined approach enhances treatment efficacy, addressing both the immediate surgical needs and the long-term management of the disease.

## Ethics and consent

Ethical approval and consent were not required.

## Consent to publish

Written informed consent for publication of their clinical details and clinical images was obtained from the patient.

**Corresponding author**

Correspondence to Habib Bessrour.

## Data Availability

No data are associated with this article.
