# Supplementary material for: Case Report: Large mediastino-abdominal hydatid cyst extending through the Larrey's hiatus: A rare case report
Source: F1000Res. 2024 Dec 5;13:1484. [Version 1] doi: 10.12688/f1000research.159463.1 (PMC11803381; doi:10.12688/f1000research.159463.1)
Supplement: Peer review history

# Reviewer report: Reviewer response for version 1

**DOI:** 10.5256/f1000research.175188.r348545

**Published:** 2024-12-30

**Authors:** Yildiray Daduk

**Affiliations:** 1 Ministry of Health Adiyaman Training and Research Hospital, Adiyaman, Turkey

1. **Detailed Explanation of the Diagnostic Process**
  - **Serological Tests:** While the article mentions that serological tests were conducted, it does not specify which tests were performed (e.g., echinococcal agglutination test, ELISA). Serological positivity is not always expected in Type IV hydatid cysts. Therefore, whether a positive result might have stemmed from another active cyst or a false positive is possible should be discussed. Was the test repeated for control purposes?
  - **Ultrasonography Findings:** Ultrasonography findings are not included in the report. Details such as the internal structure of the cyst (e.g., septation, wall thickness, calcification) and its relationship with surrounding tissues should be provided. If possible, the ultrasound image should be included.
  - **CT Findings:**
    - The anatomical features of the cyst (e.g., wall structure, fluid content, septation) should be described.
    - Mediastinal complications (e.g., pericardial effusion, compression of the trachea or major vessels) should be evaluated.
  - **Splenic Calcification:** Even if the cyst in the spleen is assumed to be inactive, this suggests potential hydatid disease involvement in other areas of the body. Further diagnostic methods (e.g., whole-body CT/MRI scans) should be employed to investigate cysts in other organs, and the results should be reported. If these were not performed, the rationale should be explained.
  - **Hiatus and Gastroscopy:** The article states that the cyst extended into the mediastinum via Larrey's hiatus. It should be clarified whether methods like gastroscopy were employed to anatomically assess the hiatus opening. If not, the reasoning should be provided.
  - **Figure Descriptions:** Figure descriptions are insufficient.
2. **Surgical Method and Rationale**
  - **Rationale for Surgical Intervention:** It is generally known that Type IV hydatid cysts do not typically require surgery. However, surgical intervention was chosen in this case. The rationale behind this decision (e.g., risk of complications from mediastinal extension or compression on surrounding structures) should be discussed in detail.
  - **Minimally Invasive Methods:** The article should explain why minimally invasive methods, such as laparoscopy or PAIR, were not considered. Were these methods deemed unsuitable for the patient?
  - **Hiatus Intervention:** It is not mentioned whether Larrey’s hiatus was addressed during surgery. If no intervention was made, the reasoning should be provided.
3. **Surgical Technique Details**
  - **Use of Scolicidal Solution:** The timing, method, and type of scolicidal solution used should be explained, as this is critical for treatment efficacy and complication prevention.
  - **Bile Fistula Risk:** For cysts larger than 10 cm, the risk of bile fistula increases. It should be clarified whether intraoperative tests (e.g., dye tests for bile ducts) were conducted.
  - **Removal of Germinal Membranes:** The article should explain how it was ensured that all germinal membranes within the thoracic cyst were completely removed, as this is vital for treatment success and preventing recurrence.
4. **Postoperative Management**
  - **Albendazole Therapy:** Type IV hydatid cysts are generally considered inactive, and postoperative albendazole therapy is not typically required. However, a six-month treatment was administered in this case. The rationale (e.g., microscopic residues or risk of recurrence) should be clearly explained.
  - **Follow-Up Process:** The long-term follow-up method for the patient should be detailed.

Are enough details provided of any physical examination and diagnostic tests, treatment given and outcomes?

Partly

Is the case presented with sufficient detail to be useful for other practitioners?

Partly

Is sufficient discussion included of the importance of the findings and their relevance to future understanding of disease processes, diagnosis or treatment?

Partly

Is the background of the case’s history and progression described in sufficient detail?

Yes

Reviewer Expertise:

Details of diagnostic tests and treatment are incomplete. Specify serological tests, imaging findings (ultrasound, CT), and treatment rationale, including surgical decisions.The discussion lacks depth on the findings' relevance. Emphasize implications for future diagnostic or therapeutic strategies.Provide more clinical details (diagnostic process, follow-up outcomes) to enhance utility for practitioners.

I confirm that I have read this submission and believe that I have an appropriate level of expertise to confirm that it is of an acceptable scientific standard, however I have significant reservations, as outlined above.

# Reviewer report: Reviewer response for version 1

**DOI:** 10.5256/f1000research.175188.r348550

**Published:** 2024-12-27

**Authors:** Zengru Xie, Xin Wang

**Affiliations:** 1 Xinjiang Medical University, Xinjiang, China; 2 Minimally Invasive Spine Surgery and Precision Orthopedics, The First Affiliated Hospital of Xinjiang Medical University, Urumqi, Xinjiang, China

I read this case report with interest, and the authors indeed reported a case of a thoracic echinococcal cyst with rare anatomical features. I believe this contributes to our understanding of the disease, especially in demonstrating the dissemination range of echinococcosis. However, referring to the CARE principles for case reports, I noticed that it lacks some content that I am particularly interested in:

 1. The patient's susceptibility factors are hardly mentioned. We do not know about the patient's living environment, lifestyle, or whether they raised livestock. Is this patient a native or an immigrant? Epidemiological information is crucial.

 2. Did the patient only undergo a CT scan? If the report could describe and display ultrasound or MRI features, it would undoubtedly enhance the manuscript, especially in the context of extensive involvement. I believe it is necessary to conduct a thorough examination of all body organs, including the bones.

 3. The authors need to inform us through intraoperative observations whether the cysts originating from the liver had any implantation or adhesion phenomena in the diaphragm, pericardium, etc., or if they were merely "transient." Did the liver also show signs of internal spread? Most liver echinococcal cysts tend to have internal metastasis; what makes this patient special? What considerations do the authors have regarding this?

 4. It would be best to provide postoperative imaging findings.

Are enough details provided of any physical examination and diagnostic tests, treatment given and outcomes?

Partly

Is the case presented with sufficient detail to be useful for other practitioners?

Partly

Is sufficient discussion included of the importance of the findings and their relevance to future understanding of disease processes, diagnosis or treatment?

Partly

Is the background of the case’s history and progression described in sufficient detail?

Partly

Reviewer Expertise:

Clinical and basic research on skeletal echinococcosis

We confirm that we have read this submission and believe that we have an appropriate level of expertise to confirm that it is of an acceptable scientific standard, however we have significant reservations, as outlined above.

# Reviewer report: Reviewer response for version 1

**DOI:** 10.5256/f1000research.175188.r348544

**Published:** 2024-12-17

**Authors:** Imane Boujguenna

**Affiliations:** 1 Ibnou Zohr University Agadir, Agadir, Morocco

Summary of the Article:

 The article presents an exceptionally rare case of a hydatid liver cyst extending into the mediastinum through Larrey's hiatus. The authors provide a comprehensive account of the case, highlighting the surgical approach adopted to achieve successful treatment.

 General Impression: The article is well-written and offers valuable insights into a unique medical phenomenon. The description of the case, along with the surgical strategy, is thorough and informative. This report contributes significantly to the literature on hydatid disease by documenting a rare occurrence and offering practical surgical guidance. 

 Strengths:

 - Originality: The documentation of the first case of a hydatid liver cyst extending into the mediastinum through Larrey's hiatus adds originality and novelty to the medical literature.

 - Clarity and Detail: The case description is clear and detailed, providing readers with a comprehensive understanding of the patient’s condition and the surgical approach taken.

 - Clinical Relevance: The emphasis on the surgical strategy offers practical insights that could benefit clinicians encountering similar cases.

 - Illustrations: The inclusion of imaging studies and surgical photographs (if applicable) enhances the reader's comprehension and provides visual evidence supporting the text.

 Suggestions for Improvement:

 - Literature Review: A more extensive review of existing literature on similar cases or related conditions could strengthen the context and significance of this case report.

 - Discussion on Alternatives: Including a discussion on potential alternative surgical strategies and their associated risks and benefits could add depth to the surgical approach section.

 - Post-operative Follow-up: Detailed information on the patient's post-operative follow-up and long-term outcomes would provide a more complete picture of the treatment’s effectiveness.

 Conclusion: This article is a valuable addition to the field of medical literature on hydatid liver cysts. The documentation of such a rare case and the detailed surgical strategy are commendable. With minor improvements, this case report has the potential to serve as a critical reference for clinicians and surgeons dealing with similar medical conditions.

Are enough details provided of any physical examination and diagnostic tests, treatment given and outcomes?

Yes

Is the case presented with sufficient detail to be useful for other practitioners?

Yes

Is sufficient discussion included of the importance of the findings and their relevance to future understanding of disease processes, diagnosis or treatment?

Partly

Is the background of the case’s history and progression described in sufficient detail?

Yes

Reviewer Expertise:

pathology, histology, cancer, biopathology

I confirm that I have read this submission and believe that I have an appropriate level of expertise to confirm that it is of an acceptable scientific standard.
